# Pervasive satellite cell contribution to uninjured adult muscle fibers

**DOI:** 10.1186/s13395-015-0067-1

**Published:** 2015-12-14

**Authors:** Bradley Pawlikowski, Crystal Pulliam, Nicole Dalla Betta, Gabrielle Kardon, Bradley B. Olwin

**Affiliations:** Department of Molecular, Cellular and Developmental Biology, University of Colorado, 347 UCB, Boulder, CO 80039 USA

**Keywords:** Satellite cell, Skeletal muscle, Pax7, Homeostasis

## Abstract

**Background:**

Adult skeletal muscle adapts to functional needs, maintaining consistent numbers of myonuclei and stem cells. Although resident muscle stem cells or satellite cells are required for muscle growth and repair, in uninjured muscle, these cells appear quiescent and metabolically inactive. To investigate the satellite cell contribution to myofibers in adult uninjured skeletal muscle, we labeled satellite cells by inducing a recombination of LSL-tdTomato in Pax7^CreER^ mice and scoring tdTomato+ myofibers as an indicator of satellite cell fusion.

**Results:**

Satellite cell fusion into myofibers plateaus postnatally between 8 and 12 weeks of age, reaching a steady state in hindlimb muscles, but in extra ocular or diaphragm muscles, satellite cell fusion is maintained at postnatal levels irrespective of the age assayed. Upon recombination and following a 2-week chase in 6-month-old mice, tdTomato-labeled satellite cells fused into myofibers as 20, 50, and 80 % of hindlimb, extra ocular, and diaphragm myofibers, respectively, were tdTomato+. Satellite cells contribute to uninjured myofibers either following a cell division or directly without an intervening cell division.

**Conclusions:**

The frequency of satellite cell fusion into the skeletal muscle fibers is greater than previously estimated, suggesting an important functional role for satellite cell fusion into adult myofibers and a requirement for active maintenance of satellite cell numbers in uninjured skeletal muscle.

## Background

Adult stem cells, which maintain and repair adult tissues, either cycle infrequently or are maintained in G_0_ [1]. Muscle stem cells, called satellite cells, reside on myofibers in a well-defined niche below the basal lamina. Satellite cells comprise only 2–11 % of the total muscle nuclei but are indispensable for normal muscle development and function [2]. Genetic ablation of satellite cells from adult mice abrogates muscle regeneration [3]. Further, satellite cell dysfunction is a component of many muscle disorders including muscular dystrophy, cancer cachexia, and age-related muscle wasting [4].

During development and in postnatal growth, myoblasts contribute extensively to skeletal muscle fibers concomitant with skeletal muscle growth. Upon reaching adulthood, skeletal muscle myofiber size and muscle mass remain relatively constant until old age (20+ months mouse) but myofiber size is dynamic, readily adapting to changing functional needs. Satellite cells, reported to be largely quiescent in adult muscle, were assumed to contribute minimally to adult muscle fibers unless required for injury repair or for hypertrophic growth. However, satellite cell contribution to myofibers appears more extensive than previously appreciated. In sedentary adult mice, up to 30 % of myofibers in uninjured hindlimb muscles acquired satellite over a 2-week interval, with satellite cell fusion varying among individual muscles [5].

Decreases in satellite cell fusion may potentiate age-related muscle wasting and exacerbate disease phenotypes. For example, satellite cell numbers decline with age, and their ability to maintain quiescence and self-renewal is compromised, all of which are likely to contribute to age-related muscle wasting [6]. However, genetic ablation of satellite cells exacerbates age-related muscle wasting only in specific muscles [7]. Given the unexpected high contribution of satellite cells to muscle during a 6-month lineage tracing experiment in adult mice, we revisited questions that have not yet been adequately addressed in uninjured skeletal muscle: first, we determined at what age satellite cell fusion into myofibers reaches steady state; second, we asked whether fusion of satellite cells into myofibers in different muscle groups varies by muscle or with mouse age; and third, we asked whether satellite cells divide prior to fusion with the myofiber.

Satellite cell fusion into skeletal muscles reaches steady state later in age than previously reported by BrdU incorporation with some muscles never reaching a steady state. Satellite cell fusion into myofibers during a 2-week lineage labeling period can occur either following cell division or directly without an intervening division and varies with mouse age. Thus, a significant functional role for satellite cell fusion in skeletal muscle homeostasis appears likely.

## Methods

### Mice

Mice were housed in a pathogen-free environment at the University of Colorado at Boulder. The University of Colorado Institutional Animal Care and Use Committee (IACUC) approved all animal protocols. *Pax7*^*Cre*^ mice [3] were crossed with ROSA-lox-stop-lox tdTomato (lsl-tdTomato) mice [8] to generate *Pax7*^*Cre*^*; R26R*^*tdTomato*^*.* Recombination was induced by five daily intraperitoneal injections of tamoxifen (Sigma-Aldrich) in corn oil dosed at 2 mg tamoxifen/20 g mouse weight beginning at 4, 8, 12, or 27 weeks of age. Tissues were collected from mice either on the last day or 2 weeks after the final dose of tamoxifen. For EdU incorporation experiments, water containing 0.5 mg/ml EdU with 1 % glucose was given to mice at the start of tamoxifen injections and maintained until collection. Wild-type mice were C57BL/6J × DBA/2J (B6D2F1/J, Jackson Labs). Mice were sacrificed by cervical dislocation prior to tissue harvest. Both male and female mice were used in this study.

### Myofiber isolation and culture

Hindlimbs were dissected, connective tissue removed, and muscle groups separated followed by enzymatic digestion in 400-U/mL collagenase at 37 °C for 1.5 h. Collagenase was inactivated by the addition of Ham’s F-12C supplemented with 15 % horse serum. Individual extensor digitorum longus myofibers were gently isolated and either immediately fixed in 4 % paraformaldehyde for 10 min (for the EdU incorporation analysis) or maintained in Ham’s F-12C supplemented with 15 % horse serum and 0.5 nM FGF-2 at 6 % O_2_ for 24 h prior to fixation and immunostaining.

### Muscle sections

Tibialis anterior, extensor digitorum longus, soleus, gastrocnemius, tongue, diaphragm, and extraocular muscles were harvested, fixed in 4 % paraformaldehyde for 2 h on ice, and incubated in 30 % sucrose overnight. Muscles were mounted for cryosectioning in sufficient O.C.T. (Tissue-Tek®) to cover the tissue. Cryosectioning was performed on a Leica Cryostat, and sections were between 8 and 12 μm. Tissues and sections were stored at −80 °C until ready for use.

### Tissue staining and immunofluorescence processing

Tissue sections were post-fixed in 4 % paraformaldehyde for 10 min at room temperature and washed three times for 5 min each in PBS prior to immunostaining. For immunostaining, both isolated myofibers and tissue sections were permeabilized with 0.2 % Triton-X100 (Sigma-Aldrich) in PBS followed by blocking with 5 % bovine serum albumin (Sigma-Aldrich) in PBS. Incubation with primary antibody occurred at 4 °C overnight followed by incubation with secondary antibody at room temperature for 1 h in 3 % bovine serum albumin in PBS. Heat-induced epitope retrieval was required to visualize Pax7 staining of muscle sections. For heat-induced epitope retrieval, post-fixed slides were placed in a citrate solution, pH 6.0, and subjected to 6 min of high pressure-cooking in a Cuisinart model CPC-600 pressure cooker. Heat-induced epitope retrieval-treated sections were incubated with 30 % H_2_O_2_ for 5 min at room temperature reduced tissue autofluorescence. To reduce non-specific binding of myosin antibodies, muscle sections were incubated with 3.6 % Mouse On Mouse Ig Blocking Reagent (MKB-2213, Vector Labs) for 1 h at 37 °C prior to antibody incubations. Antibody incubations using the Mouse On Mouse Ig Blocking Reagent were performed at 37 °C for 1 h. To visualize EdU incorporation, the Click-iT EdU Alexa fluor 488 detection kit (Molecular Probes) was used following the manufacturer’s instructions. Primary antibodies were mouse anti-Pax7, chicken anti-syndecan-4, rabbit anti-laminin (Sigma-Aldrich), and mouse IgG1 anti-MyHC IIa/IIb/IId (IIx) (clone MY-32, Sigma-Aldrich). Secondary antibodies against IgG1 or IgG of the appropriate species were conjugated to Alexa-488 or Alexa-647 (Molecular Probes) and used at 1:500. Sections and cells were incubated with 1 μg/mL DAPI for 10 min at room temperature then mounted in Mowiol supplemented with DABCO (Sigma-Aldrich) as an anti-fade agent.

### Microscopy and image processing

Myofiber images were captured on a Leica DMRXA upright spinning disc confocal microscope. Objectives were 10×/0.3 NA HC Plan Fluotar, 20×/0.7 NA HC Plan Apo, or 40×/0.85 NA HC X Plan Apo (correction collar). Leica was equipped with a Yokagawa CSU10B spinning disk, and images were taken with a Hamamatsu ImagEM EM-CCD. Muscle sections were imaged on either the Leica or Zeiss 510 LSM. Objectives used on the Zeiss were 10×/0.3 NA EC Plan Neofluar, 20×/0.8 NA Plan Apo Chromat, or 63×/1.4 NA oil differential interference contrast Plan Apo Chromat M27 lens (Carl Zeiss). Images were processed using MetaMorph Microscopy Automation and Image Analysis Software (Molecular Devices) or the FIJI ImageJ version 1.47 package (NIH) with the additional MacMaster BioPhotonics Facility plugin set. Confocal stacks were projected as maximum intensity images for each channel, background subtracted, and merged into a single image in ImageJ. Brightness and contrast were adjusted for the entire image as necessary. Images were either cropped or merged as necessary, and individual color channels were extracted without color correction or *ɣ*-adjustment. Images were adjusted and counted manually.

### tdTomato scoring

To account for the wide range of tdTomato expression in myofibers, a four-tiered scoring system was established. Myofibers brightly expressing tdTomato were scored as “1” or “+++” (see example myofiber “1” in Fig. 3c, 12 weeks). Myofibers that clearly expressed tdTomato were scored as “2” or “+” (see example myofiber “2” in Fig. 3c, 12 weeks). Myofibers that very weakly expressed tdTomato were scored as “3” or “+/−” and are counted as negative (see example myofiber “3” in Fig. 3c, 12 weeks). Myofibers that that were not brighter than the background fluorescence were scored as “4” or “−” (see example myofiber “4” in Fig. 3c, 12 weeks).

### Statistics

All statistical analyses were completed using a pairwise assessment (Student’s *t* tests). The standard error of the mean (SEM) is reported on all graphs.

## Results

### Labeling satellite cells to assess contribution to myofibers

**Fig. 1:**
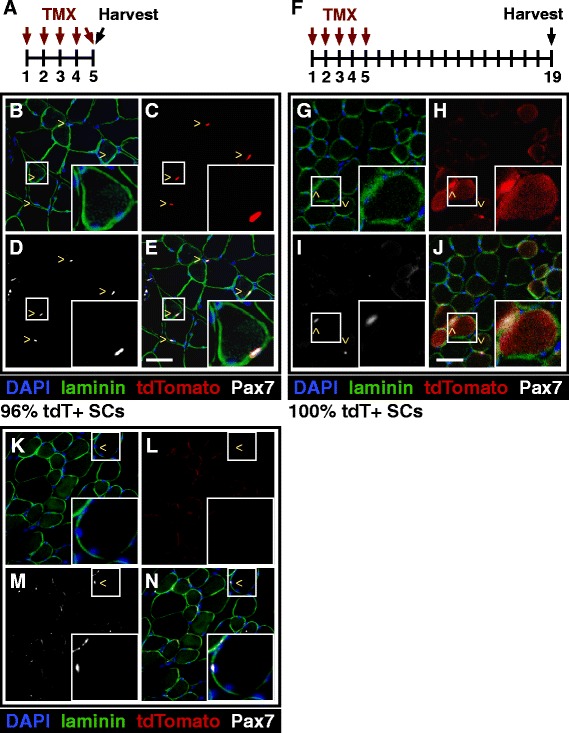
Induction of tdTomato expression in satellite cells labels myofibers by satellite cell fusion. **a** Pax7Cre;tdTomato mice were injected with tamoxifen once a day for five consecutive days to induce tdTomato expression in satellite cells. Images shown in this figure are from 6-month-old mice. **b**–**e** Representative images of TA sections show that 96 % of Pax7-expressing satellite cells are also expressing tdTomato. *Insert* highlights a Pax7+, tdTomato+ sub-laminar satellite cell. **f** Pax7Cre;tdTomato mice were injected with tamoxifen once a day for five consecutive days and then tissue was collected 2 weeks following the last injection. **g**–**j** Representative images of TA sections show Pax7-expressing satellite cells as tdTomato+ but also unexpectedly show tdTomato+ myofibers. *Insert* highlights a tdTomato-expressing myofiber with an associated Pax7+, tdTomato+ satellite cell. **k**–**n** Representative images of TA sections from Pax7Cre;tdTomato mice that were injected with only with corn oil showing no basal tdTomato expression prior to tamoxifen injection. Images display DAPI (*blue*), Sdc4 (*green*), tdTomato (*red*), Pax7 (*white*). ^ marks indicate satellite cells. Scale bars are 50 μm

We assessed the contribution of satellite cells to myofibers during postnatal growth by evaluating the extent of satellite cell fusion into postnatal myofibers in mice of various ages up to 6 months old. We labeled satellite cells by recombination with an inducible Cre in the Pax7 locus [3] and a LSL-tdTomato reporter in the ROSA locus [8]. Recombination in satellite cells was induced with five consecutive daily I.P. tamoxifen injections (Fig. 1a), and 6 h post-injection, the tibialis anterior (TA) muscle was harvested and recombination efficiency determined by scoring the percentage of Pax7 immunoreactive cells located underneath the basal lamina (Fig. 1b, e) that were also positive for tdTomato fluorescence (Fig. 1d, e; Table 1). Myofibers were tdTomato negative (Fig. 1b, e), indicating that recombination in satellite cells during the 5 days of consecutive tamoxifen injections does not promote satellite cell activation and fusion into myofibers.

**Table 1:** Quantification of tdTomato recombination

Percentage of tdTomato+ SCs	8 weeks	12 weeks	27 weeks	All time points
EDL	100.0	95.2	100.0	98.7
Sol	94.7	100.0	94.4	97.1
EOM	95.0	100.0	96.6	97.1
Gastroc	100.0	97.1	100.0	99.4
TA	96.5	97.4	100.0	97.6
Diaphragm	100.0	100.0	96.7	99.0

Cross-sections of indicated muscle with recombination induced at 8, 12, or 27 weeks were stained for Pax7, laminin, and counterstained with DAPI to mark nuclei. The number of tdTomato-expressing Pax7+ satellite cells was counted and divided by the total number of Pax7+ satellite cells. Between 20 and 80 satellite cells were counted for each experimental time point per muscle. A total of 652 satellite cells were counted. Three to six biological replicates were analyzed per time point

When TA muscles were harvested 2 weeks post-final tamoxifen injection (Fig. 1f), recombination efficiencies in satellite cells were similar to those harvested 6 h post tamoxifen injection (Table 1) where nearly all Pax7+ immunoreacitve SCs were tdTomato+ (Fig. 1g, h, j). In contrast to muscle removed 6 h post tamoxifen treatment, we observed tdTomato+ myofibers in the TA muscle at 2 weeks post tamoxifen treatment (Fig. 1h, j), where ~15 % of the myofibers were tdTomato+ (Fig. 1h, j). Neither mononuclear tdTomato+ cells (Fig. 1k–n) nor tdTomato+ myofibers (Fig. 1k–n) were observed when Pax7Cre;tdTomato mice were injected with the vehicle (corn oil) demonstrating very low or nonexistent basal recombination.

**Fig. 2:**
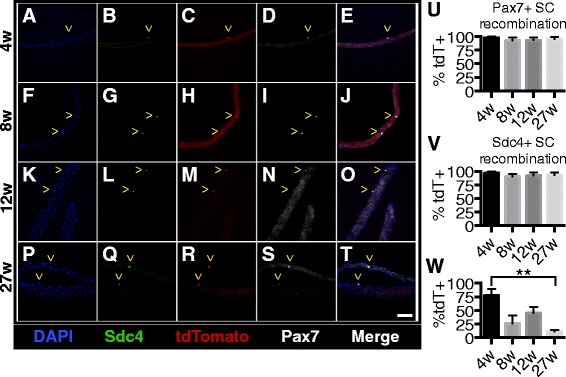
Induction of tdTomato expression does not vary with age of tamoxifen injection. **a**–**t** Images of EDL myofibers that were harvested 2 weeks after the final tamoxifen injection show that almost all satellite cells are expressing tdTomato. Images show DAPI (*blue*), Syndecan-4 (*green*), tdTomato (*red*), and Pax7 (*white*). ^ marks within micrographs indicate satellite cells. **u**–**v** Quantification showing the percentages of satellite cells on myofibers that are tdTomato+. **w** Quantification showing the percentages of myofibers that are tdTomato+. Scale bar is 50 μm. Three biological replicates were analyzed per time point

We gave five daily tamoxifen injections to mice starting at various ages (4, 8, 12 weeks, and 6 months), isolated individual extensor digitorum longus (EDL) myofibers 2 weeks post final tamoxifen injection, and then visualized tdTomato, Pax7, and Syndecan-4 24 h post-isolation initially to assess recombination frequencies. Regardless of when the tamoxifen injections were started (4 weeks (Fig. 2a–e), 8 weeks (Fig. 2f–j), 12 weeks (Fig. 2k–o), or 27 weeks (Fig. 2p–t)), almost all Pax7 immunoreactive cells were tdTomato+, with recombination efficiencies from 90 to 99 % (Fig. 2u). We verified the recombination efficiencies with an independent satellite cell (SC) marker, Syndecan-4, where all myofiber-associated SCs were Syndecan-4+, Pax7+, and tdTomato+ in myofibers isolated from mice injected at 4 weeks (Fig. 2a–e), 8 weeks (Fig. 2f–j), 12 weeks (Fig. 2k–o), and 27 weeks (Fig. 2p–t) old. Recombination frequencies were indistinguishable between myofiber-associated Pax7+ SCs (Fig. 2u) and Syndecan-4+ SCs (Fig. 2v) recombined in vivo and fixed 24 h post-isolation.

The majority of myofibers should be tdTomato+ when mice are injected with tamoxifen at 4 weeks old because satellite cells are actively contributing to growing muscle fibers. Mice injected at older ages when skeletal muscles have ceased growing should have fewer tdTomato+ myofibers. For the EDL muscle, 77 % of the isolated myofibers from mice injected at 4 weeks old were tdTomato+ (Fig. 2c, w), whereas only 12 % of EDL myofibers were tdTomato+ when isolated from mice injected at 27 weeks old (Fig. 2r, w), consistent with the cessation of postnatal skeletal muscle growth. The percentage of TdTomato+ myofibers decreased as the mouse age increased, but ~12 % of the isolated EDL myofibers from mice injected with tamoxifen at 27 weeks old were tdTomato+ when harvested 2 weeks post tamoxifen treatment.

**Fig. 3:**
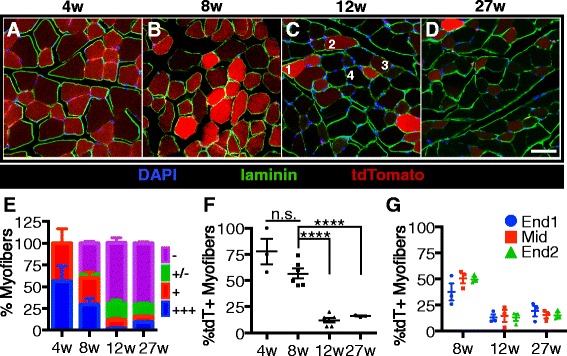
Satellite cell contribution to tibialis anterior muscle plateaus between 8 and 12 weeks of age. **a**–**d** Images of TA muscle sections from mice that were harvested 2 weeks after the final tamoxifen injection show the percentages of tdTomato expressing myofibers decrease with age. Images display DAPI (*blue*), laminin (*green*), and tdTomato (*red*). *Numbers* in the 12-week image **c** illustrate tdTomato scoring metric. **e** Quantification of tdTomato-expressing myofibers in TA muscle sections by tdTomato expression level. +++ myofibers express high levels of tdTomato (*1* in **a**), + myofibers clearly express tdTomato (*2* in **a**), +/− myofibers have very low levels of tdTomato and are scored as negative (*3* in **a**), and – myofibers do not express tdTomato at observable levels (*4* in **a**). **f** Quantification showing the percentages of myofibers that express tdTomato (+ or +++) in TA muscle sections. **g** Quantification showing the percentages of tdTomato-expressing TA myofibers by location within the muscle. Scale bar is 50 μm. ****P < 0.0001. Mean ± standard error of the mean is plotted. Significance was determined by Student’s *t* test. Three to six biological replicates were analyzed per time point

To identify the extent of labeled SC incorporation into myofibers in vivo and to determine when cessation of postnatal growth occurs, we induced recombination as described earlier (see Fig. 1f); and 2 weeks post tamoxifen treatment, hindlimb muscles were harvested, sectioned, and immunostained for laminin to identify myofibers and visualized for tdTomato. Comparing tdTomato expression in mice injected with tamoxifen at 4 weeks of age (Fig. 3a, f) to mice injected at 27 weeks old (Fig. 3d, f) reveals a major decline in tdTomato+ myofibers in hindlimb muscles at the older age. The intensity of tdTomato was variable and thus, we utilized a “1–4” grading system, scoring the most tdTomato intense myofibers at 4 (+++) and background (no detectable tdTomato) scored 1. Myofibers clearly tdTomato+ but less intense than those at 4+ were scored a 3 (++). Myofibers above background fibers but much weaker than 3 were scored a 2 (+). Examples of representative myofibers in a single field of view were labeled (Fig. 3c) and when quantified reveal the greatest numbers of intensely labeled myofibers occur in mice injected with tamoxifen at 4 weeks of age and at 8 weeks of age (Fig. 3e). In mice injected at 4 weeks of age and at 8 weeks of age, ~78 and ~60 % of the myofibers were tdTomato+, respectively (Fig. 3f). However, with the 12- and 27-week-old tamoxifen injections, the tdTomato+ labeled myofibers declined to ~20 % and were maintained at 20 %, respectively (Fig. 3f), suggesting a cessation of postnatal muscle growth in the TA muscle between 8 and 12 weeks of age. No significant differences were observed in the percentages of tdTomato+ myofibers at either muscle end compared to the midbody (Fig. 3g). The high percentage (~20 %) of tdTomato+ myofibers in TA muscles of mice injected at 27 weeks old following a brief (2 weeks) chase suggests significant SC contribution to uninjured, resting TA muscle.

### Differential satellite cell contribution among individual hindlimb muscles

**Fig. 4:**
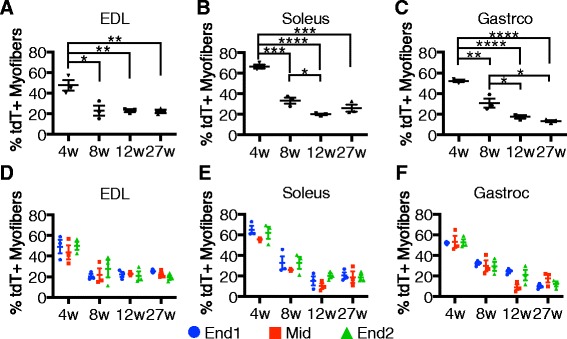
Satellite cell contribution is similar in all hindlimb muscles as a function of age. **a**–**c** Quantification of tdTomato-expressing myofibers among the EDL (**a**), soleus (**b**), and gastroc (**c**) muscles 2 weeks after the final tamoxifen injection. **d**–**f** Quantification of tdTomato-expressing myofibers by location within the EDL (**d**), soleus (**e**), and gastroc (**f**). *P < 0.05, **P < 0.01, ***P < 0.001, ****P < 0.0001. For all graphs, mean ± standard error of the mean is plotted. Significance was determined by Student’s *t* test. Three biological replicates were analyzed per time point

To determine the age when satellite cell contribution plateaus among various hindlimb muscles, tdTomato expression was analyzed in the EDL, soleus, and gastrocnemius 2 weeks after tamoxifen injection of *Pax7*^*Cre*^;LSL-tdTomato mice. In contrast to the TA muscle, the percentage of tdTomato-expressing myofibers significantly decreased in the EDL (Fig. 4a), soleus (Fig. 4b), and gastrocnemius (Fig. 4c) between the 4- and 8-week-old injections. Similar to the TA (Fig. 3f), there was a further decrease in tdTomato-expressing myofibers in the soleus (Fig. 4a) and gastrocnemius (Fig. 4c) muscles between the injections given at 8 and 12 weeks of age. The EDL was unique among the hindlimb muscles as no significant decreases in tdTomato expressing myofibers was observed in mice injected older than 8 weeks (Fig. 4a). The percentage of tdTomato+ myofibers did not significantly change in mice injected at 12 versus 27 weeks, demonstrating that all assayed hindlimb muscles reach a steady state SC fusion by 12 weeks of age. A minimum of 15 % of the labeled SCs fuse into myofibers during the 2-week chase in non-exercised, uninjured hindlimb muscle. The only muscle in which satellite cell fusion plateaus prior to 12 weeks of age is the EDL, which reaches steady state satellite cell fusion (~20 %) at 8 weeks of age. The data do not appear to be biased by the longitudinal section selected for scoring as the extent of SC incorporation at either end or at the midbody of the EDL (Fig. 4d), soleus (Fig. 4e), or gastrocnemius (Fig. 4f) was not significantly different from each other.

### Differential satellite contribution to fast and slow-twitch muscle myofibers is age-dependent

**Fig. 5:**
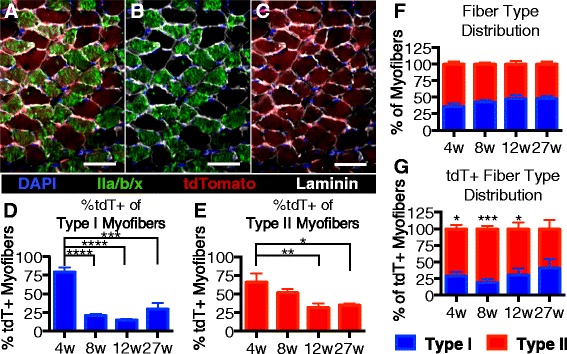
Differential contribution of satellite cells to fast and slow twitch myofibers*.* **a**–**c** Images shows soleus muscle sections from mice given tamoxifen injections at 27 weeks old and collected 2 weeks post injection. tdTomato-expressing myofibers are shown in *red*, and myofibers labeled with an antibody to identify fast twitch myofibers are shown in *green*. **d** Quantification of tdTomato expression in type I myofibers. **e** Quantification of tdTomato expression in type II myofibers. **f** Soleus fiber type distribution by age. **g** Fiber type distribution of tdTomato-expressing myofibers by age. Pseudocolored images display DAPI (*blue*), laminin (*white*), tdTomato (*red*), and fast myosin heavy chain (*green*, IIa, IIb, and IIx). Scale bars are 50 μm. *P < 0.05, ***P < 0.001. Mean ± standard error of the mean is plotted. Significance was determined by Student’s *t* test. Three biological replicates were analyzed per time point

The extent of SC fusion into adult myofibers and the age at which SC fusion plateaus was similar for all hindlimb muscles was analyzed. We selected the soleus muscle, which is comprised of ~50 % slow twitch myofibers to determine if differences were observed for SC incorporation into slow twitch (type 2) vs. fast twitch (type 1) myofibers. *Pax7*^*Cre*^*;LSL-tdTomato* mice were injected with tamoxifen at the indicated ages and the soleus muscles collected 2 weeks post final tamoxifen injection. Muscle sections were then stained with an antibody that recognizes all fast twitch muscle fibers types (Fig. 5a–c; IIa/b/x) (Havenith et al. [9]). Unlabeled myofibers were assumed to be slow twitch (Fig. 5a–c), and the percentages of fast and slow myofibers were quantified for each age (Fig. 5f). When tamoxifen was injected into 27-week-old mice, 2 weeks later, 25 % of the soleus myofibers were tdTomato+ (Fig. 4b). Further analysis of the muscle from mice injected at 27 weeks of age identified 60 % of the tdTomato+ soleus myofibers as fast twitch and 40 % of the tdTomato+ myofibers as slow twitch, (Fig. 5a–c, g), the same ratio of fast twitch to slow twitch muscle among the entire 27-week myofiber population (Fig. 5f). The contribution of satellite cells to type 1 fast twitch myofibers (Fig. 5d) plateaus at an earlier age than for type II slow twitch (Fig. 5e), indicating that satellite cell fusion rates differ for slow and fast twitch myofibers during postnatal muscle growth.

As shown in the previous figure, the percentage of tdTomato expressing myofibers in the soleus muscle decreases from 60 to 30 % in mice injected at 4 versus 8 weeks of age (Fig. 4b), yet the percentage of fast twitch tdTomato+ myofibers remains constant at approximately 40 % (Fig. 5e). Therefore, between 4 and 8 weeks of age, satellite cells contribute predominately to fast twitch myofibers (Fig. 5d). Collectively, satellite cells in the soleus preferentially contribute to fast twitch myofibers during adolescent growth and then satellite cells contribute equally to fast and slow twitch muscle in adult, once postnatal growth has ceased.

### Satellite cell contribution to the extraocular and diaphragm muscle differs from hindlimb muscles

**Fig. 6:**
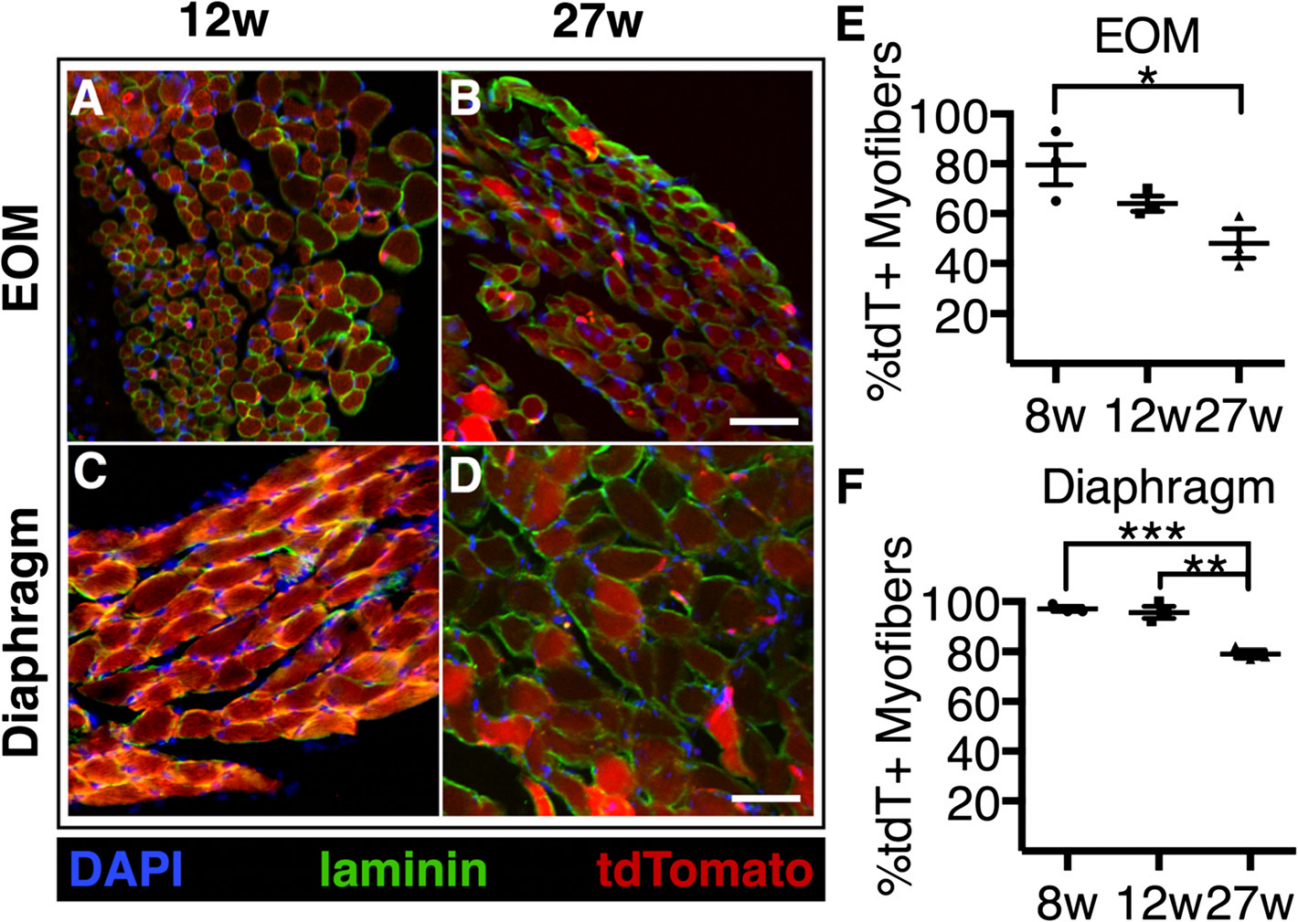
Satellite cell contribution to EOM and diaphragm muscles does not plateau with age. **a**–**d** Images show tdTomato expressing myofibers in the EOM and diaphragm 2 weeks after the final tamoxifen injection. **e**–**f** Quantification showing the percentages of tdTomato-expressing myofibers in sections of the **e** EOM and **f** diaphragm. Images display DAPI (*blue*), laminin (*green*), and tdTomato (*red*). Scale bar is 50 μm. *P < 0.05, **P < 0.01. For all graphs, mean ± standard error of the mean is plotted. Significance was determined by Student’s *t* test. Three biological replicates were analyzed per time point

The diaphragm and extraocular (EOM) muscles were selected for analysis as the diaphragm is severely affected in dystrophic mice and the EOM is spared in dystrophic mice, respectively [10]. To analyze the EOM and diaphragm muscles, mice were given tamoxifen injections at the same time points that were used in the hindlimb analysis (4, 8, 12, and 27 weeks of age). EOM and diaphragm muscles were collected 2 weeks later and scored for tdTomato+ myofibers. Satellite cell incorporation into EOM was much greater than that observed in hindlimb muscles, where 80 % of the EOM myofibers were tdTomato+ in mice injected at 8 weeks of age (Fig. 6e). Distinct from the hindlimb muscles, SC fusion into the diaphragm and EOM did not plateau, where tdTomato+ myofibers were at 64 (Fig. 6a, e) and 48 % (Fig. 6b, e) when injected at 12 and 27 weeks of age, respectively. We did not analyze older mice and do not know whether SC fusion reaches a steady state in EOM.

Satellite cell fusion into the diaphragm, similar to the EOM, does not plateau by 27 weeks of age yet is very high, where 95 (Fig. 6f), 90 (Fig. 6c, f), and 80 % (Fig. 6d, f) of the diaphragm myofibers were tdTomato+ following in mice injected at 8, 12, and 27 weeks old, respectively. Satellite contribution in adult EOM and diaphragm is significantly higher than satellite cell contributions in hindlimb muscle of mice injected at 27 weeks old.

**Fig. 7:**
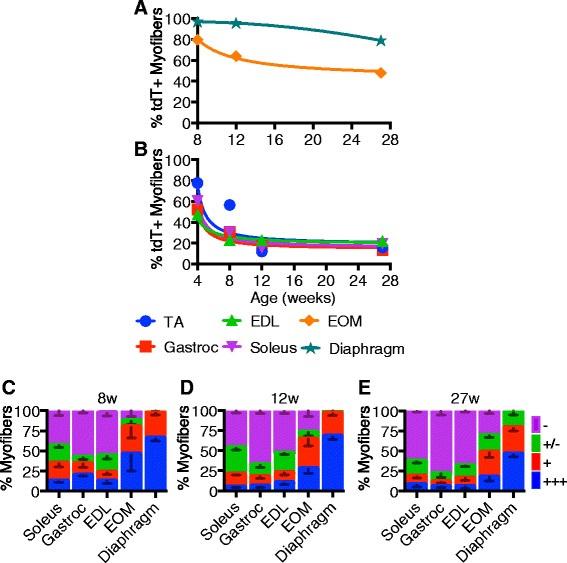
Satellite cell contribution to myofibers is age dependent and varies in different muscles. **a**–**b** Non-linear regression trend lines for percentages of tdTomato-expressing myofibers through experimental time points from the EOM and diaphragm (**a**) and EDL, soleus, and gastrocnemius (**b**). **c**–**e** Quantification of myofiber tdTomato-expression of hindlimb, EOM, and diaphragm muscles with recombination induced at 4 (**c**), 12 (**d**), or 27 weeks (**e**). Quantification of tdTomato was evaluated at four relative levels of expression: +++ very high tdTomato intensity (see example myofiber “*1*” in Fig. 3c, 12 weeks), + intermediate tdTomato intensity (see example myofiber “*2*” in Fig. 3c, 12 weeks), +/− very low tdTomato intensity myofibers and scored as negative (see example myofiber “*3*” in Fig. 3c, 12 weeks), and – fibers do tdTomato intensity indistinguishable from background (see example myofiber “*4*” in Fig. 3c, 12 weeks)

A comparison of the muscles analyzed illustrates overall similarities between the hindlimb muscles, where hindlimb muscle SC fusion plateaus by 12 weeks of age (Fig. 7b). However, the number of tdTomato+ myofibers differs among the hindlimb muscles when tamoxifen is given prior to 12 weeks of age (Fig. 7b), where the TA muscle is the last to stabilize SC fusion at 15 % at 12 weeks of age (Fig. 7b). In contrast, the diaphragm and EOM decline more linearly over time (Fig. 7a) and do not achieve steady state SC fusion with high levels of tdTomato+ myofibers present when tamoxifen is injected at 27 weeks of age. Plotting the relative intensity of tdTomato fluorescence for each muscle between the 8-, 12-, and 27-week-old injections reveals a decline in the tdTomato fluorescence intensity with increasing age until a steady state is reached in the hindlimb muscles (Fig. 7c).

### Satellite cell fusion into uninjured myofibers can occur with either an intervening cell division or without cell division

**Fig. 8:**
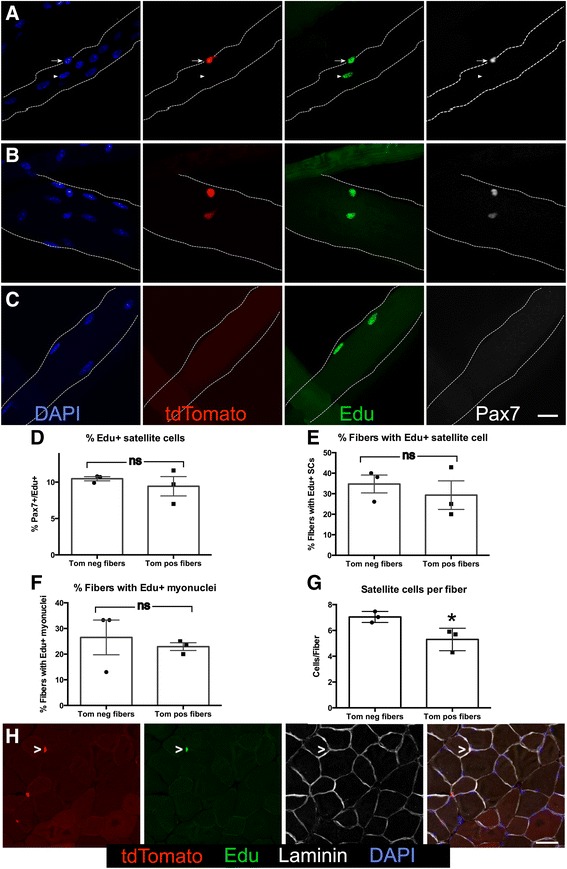
Satellite cells fuse into uninjured myofibers either with our without an intervening cell division. **a**–**c** Representative images show EdU+ satellite cells and EdU-labeled nuclei within myofibers. **a** Myofiber showing an EdU+ satellite cell (*arrow*) and an EdU+ differentiated myonuclei (*arrowhead*). **b** Images show a myofiber with two satellite cells that are EdU+. **c** A myofiber with two EdU+ myonuclei within the myofiber. For **a**–**c**, DAPI (*blue*), tdTomato (*red*), EdU (*green*), and Pax7 (*white*). Scale bar is 20 μm. **d** Quantification showing the percentages of satellite cells on dtTomato+ (*Tom pos*) myofibers versus dtTomato− myofibers (*Tom neg*) that were labeled with EdU. **e** Bar graph showing the percentages of myofibers that have at least one EdU+ associated satellite cell. **f** Quantification showing the percentages of myofibers that have at least one EdU+ nuclei within the myofiber. **g** Quantification of the satellite cell number present on dtTomato+ myofibers versus dtTomato− myofibers (*p ≤ 0.01). **h** Images of TA muscle sections show an EdU+, tomato+ SC. tdTomato (*red*), EdU (*green*), laminin (*white*), and DAPI (*blue*). Scale bar is 40 μm. For bar graphs, *ns* denotes not significant. Significance was determined by Student’s *t* test. *N* = 3. For all graphs, the mean with the standard error of the mean is plotted

EdU was added to the drinking water of 5-month-old Pax7Cre;tdTomato mice concomitant with the initiation of tamoxifen injections. Muscle tissue was collected 2 weeks following the final tamoxifen injection and individual EDL myofibers fixed immediately after isolation. Essentially, all Pax7 expressing SCs were tdTomato+, while ~12 % of the EDL myofibers were tdTomato+. Approximately 10 % of the SCs were labeled with EdU following the chase period (Fig. 8a, b, d), with ~35 % of the myofibers possessing one or more EdU+ -associated SC (Fig. 8e). Similar numbers of EdU+ SCs were present on tdTomato+ myofibers and tdTomato− myofibers (Fig. 8d, e) with about 25 % possessing one or more EdU+ myonucleus (Fig 8a, c, f). No more than two EdU+ nuclei were found associated or within any single myofiber where the percentage of myofibers possessing at least one EdU+ myonucleus did not significantly differ between tdTomato+ myofibers (23 %) and tdTomato– myofibers (27 %) (Fig. 8f). Since the majority of tdTomato+ myofibers did not possess an EdU+ nucleus (77 %) the majority of SC fusion occurs without an intervening cell division. Furthermore, EdU+ myonuclei in tdTomato− myofibers suggests that multiple SCs must fuse for visualization of tdTomato expression in myofibers. This conclusion is consistent with fewer SCs on tdTomato+ myofibers compared to the SC number on tdTomato– myofibers (Fig. 8g).

TA muscle, EOM, and diaphragm were collected from EdU-exposed recombined Pax7^CreER^;LSL-tdTomato 5 months old mice where EdU+ cells are clearly visible in the muscle tissue sections (Fig. 8h). Attempting to reliably quantify EdU+ SCs proved impractical due to the EdU uptake by other cells and the difficulty in identifying EdU+ cells following antigen retrieval necessary for Pax7 detection.

## Discussion

The majority of satellite cells in adult muscle are presumed quiescent [11] and are thought to rarely activate, proliferate, and fuse into existing myofibers [12]. Indeed, a requirement for SC contribution to myofibers in uninjured muscle, for muscle hypertrophy, and contributing to loss of muscle mass during aging has been questioned [13], yet several recent publications demonstrate satellite cell intrinsic deficits in satellite cells derived from aged muscle [14]. We utilized an inducible Pax7 Cre knock in allele [3] and a sensitive tdTomato lineage reporter [8] to assess the extent of satellite cell contribution to a number of skeletal muscles spanning juvenile mice undergoing active postnatal muscle growth to an adult 6-month-old mouse. We asked whether satellite cells divide prior to their fusion into myofibers in uninjured muscle and asked when fusion homeostasis is acquired by assessing the extent of Pax7+ cell fusion into myofibers as a function of age in different muscles. Since satellite cells are derived from the Pax7 expressing cell population in the postnatal mouse, the number of tdTomato+ satellite cells fusing into existing muscle fibers will yield myofibers of different tdTomato+ intensities. A 5-day tamoxifen dose regimen was sufficient to label nearly all detectable Pax7+ satellite cells with little or no detectable tdTomato+ myofibers. However, following a 2-week chase, we consistently observed tdTomato+ myofibers and thus, used this time point to assess the extent of satellite cell fusion. TdTomato+ myofibers were observed at all ages, suggesting substantive satellite cell fusion and turnover as satellite cell numbers remain constant in adult mice from 3 to 6 months of age. Consistent with this observation, 10 % of SCs were EdU+ following 19 days of EdU exposure. TdTomato+ cell incorporation varied between distinct muscles, with the satellite cell contribution to all hindlimb muscles plateauing by 12 weeks of age. Neither the diaphragm nor the extraocular muscles achieved a steady state level of tdTomato+ cell incorporation, indicating that the satellite cell fusion into these muscles does not plateau even by 6 months of age.

All hindlimb muscles examined reached a steady state level of cell fusion by 12 weeks of age, contrasting with prior methods used to assess the extent of satellite cell fusion in the mouse [15]. The level of SC fusion is unexpectedly high as ~20 % of the myofibers in hindlimb muscles appear tdTomato+ following a 2-week interval post recombination. Since SC numbers remain constant, SCs must proliferate to replenish the SCs lost to cell fusion. Myonuclei remain constant as well [15] and thus, significant turnover of SCs and myonuclei is likely to occur, unless myonuclei accumulate at the myotendonous junctions, where they are not routinely counted. Although SC fusion and myonuclear turnover/accretion in hindlimb muscles are a small fraction of the total numbers of myonuclei, a much higher percentage of new myonuclei is acquired by fusion of SCs into the diaphragm and EOM. Thus, these skeletal muscles appear to be undergoing continuous turnover of myonuclei, consistent with prior published observations SC incorporation and myonuclear turnover in the EOM [16]. SC incorporation and myonuclear turnover have not been examined in the diaphragm muscle. The extent of SC incorporation does not appear to correlate with sparing from muscular dystrophies as the EOM is generally spared [10] and the diaphragm is severely affected [17].

In hindlimb muscles, the function of SC fusion and inferred myonuclear turnover is incompletely clear as the effects of satellite cell depletion using Pax7-driven diphtheria-toxin A have minimal reported effects on muscle mass, myofiber size, or myonuclear number in adult mice [13] and no exacerbation of age-related muscle loss [7]. The latter conclusion is inconsistent, however, with a more recent examination of muscle loss following SC ablation in adult uninjured mice where a decrease in the size of myofibers was observed in the EDL [5]. We observed a greater than expected extent of SC fusion, where nearly all myofibers incorporate satellite cells during the growth phase (8 weeks and younger), reaching a plateau in all hindlimb muscles between 8 and 12 weeks of age. Nevertheless, the predicted turnover of the SC pool and myonuclear pool, assuming these pools remain relatively constant, is surprisingly high and thus, likely to play a functionally important role despite claims that SCs are not required for skeletal muscle hypertrophy or to maintain skeletal muscle mass [13].

Data from a relatively short EdU labeling period (19 days) is consistent with the extent of SC fusion as 10 % of SCs are EdU+ and consistent with recent data reporting that a significant percentage of SCs incorporate BrdU or dilute expression of a lineage marker over a period of several weeks [6]. Unexpectedly, SCs appear to fuse into an uninjured myofiber without an intervening cell division. This observation is consistent with symmetric SC division observed by EdU incorporation (Fig. 8) and induced by wnt7a (LeGrand et al. [18]). In addition to symmetric SC division, we observed myofibers possessing two neighboring EdU+ myonuclei (Fig. 8c) likely to arise from symmetric SC division followed by symmetric differentiation. Finally, asymmetric SC divisions were observed on uninjured myofibers where one SC differentiates and fuses, while the other daughter retains an SC phenotype consistent with myofibers possessing one EdU+ myonuclei and one EdU+, Pax7+ SC (Fig. 8b). Although we cannot definitively conclude that SCs underwent symmetric and asymmetric division following EdU incorporation, the observations that (1) no more than two EdU-labeled nuclei were observed following myofibers isolation, (2) the myofibers were fixed immediately upon isolation, and (3) the EdU+ “daughters” were always in close proximity is consistent with these “daughters” arising from SC division.

The numbers of SCs associated with fast and slow twitch muscles differ [19], but we observed no differences in fusion of SCs with fast or slow twitch myofibers once steady state levels of satellite cell fusion were achieved, suggesting that establishment of SC numbers is actively regulated. Active regulation of SCs likely differs between muscles as we observed large differences for SC incorporation that vary by muscle and by mouse age. Differential satellite cell contribution among different muscle groups may reflect heterogeneity among satellite cells or differences in SC regulation. In support of the former possibility are gene expression profiles examining satellite cells from EOM and pharyngeal muscle, EDL and masseter muscle, revealing distinct genetic programs [20–22] as well as distinct transplantation efficiencies for SCs derived from the EDL, soleus, masseter, and TA muscles [22, 23]. A thorough understanding of the extent of SC fusion and analyses of myonuclear turnover is needed to understand the function of continuous satellite cell incorporation occurring in uninjured skeletal muscle.

## Conclusions

Lineage labeling of SCs followed by a 2-week chase revealed significant incorporation of satellite cells into myofibers of all muscles examined, with nearly all myofibers incorporating SCs in 4-week-old mice. SC fusion plateaued to ~20 % in the hindlimb muscles by 12 weeks of age, persisting at 20 % through 27 weeks of age. Although prior data suggest SCs are quiescent in adult uninjured muscle, a significant percentage divide and fuse as myonuclei. Fusion occurs with or without an intervening cell division, requiring SC expansion to maintain a constant SC pool. SC fusion varied with individual muscles as SC incorporation did not plateau in 6-month-old EOM and diaphragm muscles where 50 and 80 % of the myofibers were tdTomato+, respectively. SC fusion into skeletal muscle fibers occurs much more extensively than previously reported, suggesting an important functional role for SC fusion into adult myofibers and a requirement for active maintenance of SC numbers in uninjured skeletal muscle.

## Abbreviations

SC: Satellite Cell
TA: tibialis anterior
EDL: extensor digitorum longus
Sol: Soleus
EOM: extra ocular muscle
Gastroc: gastrocnemius
EdU: 5-ethynyl-2′-deoxyuridine
